# Canadian aging and inactivity study: Spaceflight-inspired exercises during head-down tilt bedrest blunted reductions in muscle-pump but not cardiac baroreflex in older persons

**DOI:** 10.3389/fphys.2022.943630

**Published:** 2022-09-21

**Authors:** Farshid Sadeghian, Donya Naz Divsalar, Rabie Fadil, Kouhyar Tavakolian, Andrew P. Blaber

**Affiliations:** ^1^ Department of Biomedical Physiology and Kinesiology, Aerospace Physiology Laboratory, Simon Fraser University, Burnaby, Canada; ^2^ Biomedical Engineering Program, University of North Dakota, Grand Forks, ND, United States

**Keywords:** muscle-pump baroreflex, exercise countermeasure, postural hypotension, aging, cardiac baroreflex

## Abstract

As part of the first Canadian aging and inactivity study (CAIS) we assessed the efficacy of space-based exercise countermeasures for maintenance of cardiac and muscle-pump baroreflex in older persons during bedrest. An initiative of the Canadian Space Agency, Canadian Institutes of Health Research and the Canadian Frailty Network, CAIS involved 14 days of 6-degree head-down tilt bedrest (HDBR) with (Exercise) or without (Control) combined upper and lower body strength, aerobic, and high-intensity interval training exercise countermeasures. Twenty healthy men and women aged 55 to 65, randomly divided into control and exercise groups (male control (MC, *n* = 5), male exercise (ME, *n* = 5), female control (FC, *n* = 6), female exercise (FE, *n* = 4)) (age: 58.7 ± 0.5 years, height: 1.67 ± 0.02 m, body mass: 70.2 ± 3.2 kg; mean ± SEM), completed the study. Cardiac and muscle-pump baroreflex activity were assessed with supine-to-stand tests. Wavelet transform coherence was used to characterise cardiac and muscle-pump baroreflex fraction time active (FTA) and gain values, and convergent cross-mapping was used to investigate causal directionality between blood pressure (BP) and heart rate, as well as BP and lower leg muscle electromyography (EMG). Seven of the twenty participants were unable to stand for 6 minutes after HDBR, with six of those being female. Our findings showed that 2 weeks of bedrest impaired skeletal muscle’s ability to return blood to the venous circulation differently across various sexes and intervention groups. Comparing values after bed rest with before bed rest values, there was a significant increase in heart rates (∆ of +25%; +17% in MC to +33% in FC; *p* < 0.0001), beat-to-beat EMG decreased (∆ of −43%; −25% in ME to −58% in MC; *p* < 0.02), while BP change was dependent on sex and intervention groups. Unlike their male counterparts, in terms of muscle-pump baroreflex, female participants had considerably decreased FTA after HDBR (*p* < 0.01). All groups except female control demonstrated parallel decreases in cardiac active gain and causality, while the FC demonstrated an increase in cardiac causality despite a similar decline in cardiac active gain. Results showed that the proposed exercises may alleviate muscle-pump baroreflex declines but could not influence the cardiac baroreflex decline from 14 days of inactivity in older adults.

## Introduction

The elderly population is increasing globally (Vandewoude et al., 2012), and it is predicted that the number of individuals over the age of 60 will surpass two billion by 2050 (Wang et al., 2016). Aging is often characterized by a decrease in physical activity and an increase in sedentary behavior. Both of these changes in lifestyle aggravate the lack of functionality in physiological systems (Mechling and Netz, 2009), which can result in deterioration of overall functional health, such as through orthostatic hypotension (Chodzko-Zajko et al., 2009; Lanier et al., 2011). Concerns about increased rates of sedentary lifestyles in older people heightened with the imposition of limits to personal movements during the Covid-19 pandemic to reduce viral transmission. Research has also shown adaptations of physiological systems during spaceflight to be similar to aging (Convertino et al., 1989; Convertino et al., 1990; LeBlanc et al., 2007; Platts et al., 2009; Spector et al., 2009).

Spaceflight-induced weightlessness is known to reduce muscle size and strength and cause functional changes of the heart and blood vessels which alters circulating blood and interstitial fluid volumes, arterial blood diastolic pressure, ventricular stroke volume, left ventricular mass and, resetting of the carotid baroreceptors (Antonutto and Di Prampero, 2003; Goswami et al., 2013). These multi-system changes can negatively affect the astronaut’s ability to perform mission-related tasks and increase the risk of loss of consciousness and fainting upon re-introduction to gravity (e.g., landings on the moon or Mars). Head-down tilt bedrest (HDBR), similar to space flight, removes the gravitational hydrostatic pressure created by standing and the stresses of standing and walking from the musculature, and can simultaneously decondition the cardiovascular and skeletomuscular systems. Thus, HDBR is a validated technique for simulating microgravity exposure, which enables us to track the changes in the relationship between these systems, especially the relationship of BP to muscular activation.

Since the early days of human spaceflight, physical exercise has been highlighted as a potential countermeasure to cope with weightlessness-induced deconditioning. The process of space adaption appears to be similar to those found with prolonged inactivity (Moore et al., 2010). Knowledge from the implementation of space-based countermeasures can provide important insight for those interested in medicine and rehabilitation. During space missions, an effective, multi-purpose, and non-invasive countermeasure for preserving muscles and cardiovascular components is essential. A detailed examination of these and their history in spaceflight and bedrest are presented in depth by Hedge et al. (Hedge et al., 2022).

On board the international space station astronauts use three different types/modalities of exercise equipment; cycle ergometer, treadmill, and advanced resistive exercise device (ARED) (Laws et al., 2020). Each exercise has a distinct purpose. Astronauts have relied on cycle ergometer exercising since the early days of spaceflight for a variety of reasons, including its ability to accurately measure work output by systematically altering pedaling resistance and for its benefits to the cardiovascular system (Convertino, 1996; Laws et al., 2020). Walking or jogging on the treadmill is also the most crucial factor in maintaining bone and muscle health as it can generate impact forces on body (Swain et al., 2021). Finally, the ARED offers a multi-purpose whole-body workout that includes back squat, sumo squat, sumo deadlift, shrugs, shoulder press, bench press, bicep curl, triceps extension, and single-arm row (Laws et al., 2020).

Understanding spaceflight-induced changes in the body (e.g., cardiovascular deconditioning and loss of skeletal muscle mass) are not just important for improving astronaut health; they could also contribute to the development of countermeasures and therapies that help people suffering from age-related conditions and diseases on Earth. Although space countermeasures are not necessarily appropriate for the elderly on Earth, as they are designed for relatively healthy and fit individuals, they can help geriatricians and rehabilitation specialists gain a better understanding of musculoskeletal and cardiovascular alterations and to establish a treatment/prevention program (Goswami et al., 2012; Goswami et al., 2013). Furthermore, age-related physiological changes are linked to hormones, exercise levels, diet, and illness, making it difficult to pinpoint the root causes of muscle loss and cardiovascular changes (Clément, 2011). Exploring the relationship between spaceflight countermeasure use and aging would thus shed insight on the aging process and give unique viewpoints and innovative techniques for incorporating into Earth medicine and rehabilitation. (Goswami et al., 2012; Blaber et al., 2013; Goswami et al., 2013).

Bedrest, which is best characterized by immobilization and confinement, has acted as an informative analogue to investigate the impact of inactivity on musculoskeletal and cardiovascular systems. It has been shown that bedrest in healthy older individuals can result in a reduction of muscle size and strength, as well as changes in the function of the heart and blood vessels. Previous literature has shown that only 10 days of bedrest in older persons induced remarkable muscle weakening including a loss in whole-body lean mass (−1.50 kg; *p* = 0.004), lower extremity lean mass (−0.95 kg; *p* = 0.003), and strength (−19 Nm s^−1^; ∆ of −15.6%; *p* = 0.001) which is significantly greater than seen annually in the average aging population (Kortebein et al., 2007). Furthermore, six-degree head-down bedrest (HDBR) has been shown to be an effective analogue of microgravity/spaceflight conditions to simulate cardiovascular and musculoskeletal systems’ deconditioning (Goswami et al., 2015; Goswami, 2017). Due to limited resources for human spaceflight research, prolonged HDBR serves as an ideal experimental environment to study post-flight deconditioning in astronauts.

In this research, we investigated the impact of combined upper and lower body strength, aerobic, and high-intensity interval training (HIIT) exercise countermeasures designed for older persons (Hedge et al., 2022) on maintaining the cardiac and muscle-pump baroreflexes in healthy 55–65 year old men and women during 14 days of 6-degree head-down tilt bedrest (HDBR). This research was conducted as part of the Canadian aging and inactivity study (CAIS), supported by the Canadian Institutes of Health Research (CIHR), Canadian Frailty Network (CFN), and the Canadian Space Agency (CSA). In this paper we explore the relationship between biological sex and exercise intervention (four separate cohorts including males and females in both control and exercise groups) on the physiological interplay between the cardiovascular and musculoskeletal systems for blood pressure (BP) regulation. Previous research showed that both systems were severely impacted by bedrest following 60 days of HDBR without exercise in middle-aged males (Xu et al., 2020).

Our team has developed a series of techniques to study the significance of lower limb muscle activity in maintaining BP. For this purpose, we adapted the wavelet transform coherence (WTC) analysis (Garg et al., 2013; Garg et al., 2014; Xu et al., 2017) and convergent cross mapping (CCM) causality (Verma et al., 2017a; Verma et al., 2017b; Verma et al., 2018) methods to extract indices that characterize the interaction time (fraction time active, FTA), response gain value (gain), and control directionality (causality) among cardiovascular and postural measurements. We hypothesized that daily activation of the muscles associated with both posture and the muscle-pump would limit the decline in the muscle-pump blood pressure reflex in terms of coupling (causality), strength (gain), and activity (FTA). Similarly, it was expected that aerobic exercise would positively affect the cardiac baroreflex.

## Materials and methods

### Study design and testing protocols

**FIGURE 1:**
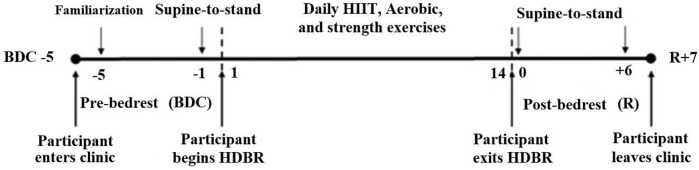
Timeline—the participants remained at the testing facility for a total of 26 days, of which 14 days were spent in 6° head-down tilt bedrest (HDBR). Participants arrived at MUHC 5 days prior to entering HDBR. At this time baseline data collection (BDC) was performed. After bedrest, participants remained at the clinic for 7 days where recovery (R) data were collected. A familiarization StS was performed on BDC-5 followed by research StS tests in the mornings of BDC-1 and R+0, R+6.

This experiment was conducted at the Center for Innovative Medicine (CIM) of the McGill University Health Centre Research Institute (RI-MUHC). The study consisted of four 26-days bedrest campaigns (Figure 1), during which 5 to 6 participants per campaign were subjected to HDBR at −6° to simulate spaceflight-induced fluid shifts.

Half of the participants received an exercise countermeasure procedure during the HDBR, while the other half served as controls and received stretch and joint movement physiotherapy. Daily exercises consisted of a combination of three sessions of the following: HIIT, low-intensity aerobic activity, and lower-body strength exercises, resulting in forty-two exercises over the 2 weeks of HDBR with 60 min of daily physical activity (Table 1). A detailed description of the exercise protocols is provided by Hedge et al. (Hedge et al., 2022) along with the rationale for their implementation. Briefly, like in-space exercise programs, cycling and resistive training regimes along resistive bands were prescribed for preserving muscles and cardiovascular health (Hedge et al., 2022). In addition, HIIT exercise was incorporated into the bedrest exercise program (Hedge et al., 2022). Equipment was modified so that all workouts were conducted in a head-down tilt posture. The intensity of the exercise countermeasures was modified individually according to the participants’ performance and tolerance, as measured by heart rate and BP throughout HDBR. Apart from the exercise sessions, there were no differences in standards of care between the two groups. Food intake was prescribed and monitored by the MUHC staff based on the nutritional requirements for the control and exercise groups as well as by biological sex (Supplementary Material). Participants were provided with liquids as part of their regulated diet and were allowed water hydration *ad libitum*.

**TABLE 1:** Bedrest exercise protocols. A combination of up to three per day were performed with a maximum total time of 62 min per day.

Exercise	Type	Duration	Intensity	Total
Lower strength	Body weight, cables, resistance bands	25 min	12 times–max tolerance*	4
Upper strength	Body weight, cables, resistance bands	25 min	12 times–max tolerance*	5
Aerobic	HIIT	32 min (30 s on, 90 s off)	80–90% HRR	7
Aerobic	Progressive	15 min	30–60% HRR	14
Aerobic	Continuous	15 min	60–70% HRR	6
Aerobic	Continuous	30 min	60–70% HRR	6

Legend: *: By asking participants during each set; HRR: Heart rate reserve (max HR, resting HR, max HR, measured prior to bedrest).

Overall ethical approval for CAIS was obtained from the research ethics board of the MHUC. The study was registered as a clinical trial (NCT04964999: Microgravity Research Analogue (MRA): Understanding the Health Impact of Inactivity for the Benefit of Older Adults and Astronauts Initiative) in the US National clinical trial registry. Research and data collection associated with our component of the study was approved by the Office of Research Ethics at Simon Fraser University. The participants signed a written informed consent and agreed to be available at MHUC for the entire 26 days study period. The research was conducted in compliance with the guidelines and regulations of the above agencies and the declaration of Helsinki.

### Data collection

An electrocardiogram (ECG) was recorded with a bipolar three-lead ECG (IX-BIO4, iWorx, United States) in a standard Lead II electrode configuration. The non-invasive Portapres (FMS, Amsterdam, Netherlands) was used to monitor continuous BP at the finger, with absolute BP height-corrected to the heart level. Surface EMG was recorded transdermally from four bilateral lower leg muscles, including the tibialis anterior, lateral soleus, and medial and lateral gastrocnemius, using the Bagnoli-8 (Delsys Inc., MA, United States) EMG system. The SENIAM project’s (Hermens et al., 1999) suggestions were used to select the locations for EMG sensor placement. Data were collected at 1,000 Hz using a National Instruments USB-6218 16-bit data capture equipment and LabVIEW 2013 software (National Instruments Inc., TX, United States).

### Supine-to-stand test procedure

The phases of the study included 5 days of baseline data collection (BDC), 14 days of HDBR, and 7 days of recovery (R) (Figure 1). A supine-to-stand (StS) test was administered to activate and assess the cardio-postural control system (Blaber et al., 2009; Garg et al., 2013; Garg et al., 2014; Verma et al., 2017a; Rodriguez et al., 2017; Xu et al., 2017) twice during BDC and twice on recovery days. StS tests were performed in the mornings of BDC-5, BDC-1, R+0, R+6 (Figure 1). As the participants had not conducted an StS test in the screening process, the initial test on BDC-5 was considered a familiarization protocol for the participant. It should be noted that StS tests performed on BDC-1 and R+0 and were conducted 1 hour after the Canadian Space Agency (CSA) standard tilt test, which had a maximum duration of 15 min.

A room with no windows in a silent location was selected for the StS test to ensure participants’ deprivation of auditory and visual stimuli during the protocol. Upon arrival at the testing room, the participants were placed in a supine position and instrumented for physiological monitoring. After instrumentation, lights were turned off and the participants were instructed to close their eyes while continuous data acquisition took place for 5 min. Following this, participants were asked to open their eyes and were assisted to the standing position. One researcher would sweep their legs off the bed, and another would assist with raising their torso. Participants’ feet were placed parallel and 5 cm apart during standing. During the subsequent 6-min of quiet stance, they were instructed to keep their eyes closed with their arms relaxed at their sides, maintain an imaginary eye-level gaze, and not alter foot placement (Redfern et al., 2007).

### Data analysis

In this study, we report the results from the stand portion of the StS test. The minute of data related to going from supine to stand was not utilized because of the existence of movement disturbance during the transition phase. At the end of this minute when the participant had their feet in the proper position, they were facing directly forward, and were standing free of assistance, the stand clock was started. We analysed the first 180 s of the stand to examine the reflex responses immediately following the transition period. The data analysis process has been previously elaborated in detail by Xu et al. (Xu et al., 2017). In summary, The ECG signal was used to calculate RR intervals. The maximum and minimum values in the BP waveform during a heartbeat were used to determine beat-to-beat SBP and diastolic blood pressure (DBP). Mean arterial pressure (MAP) was computed as the average BP from end-diastole to end-diastole of the waveform. Individual muscle beat-to-beat EMG (EMG impulse) was determined as the mean area under the rectified EMG envelope between successive heartbeats. The rectified EMG recorded from four separate muscles in each leg was summed to depict total muscle activity in the form of aggregate EMG. Before wavelet transform coherence and causality analyses, beat-to-beat physiological signals were interpolated using the spline approach and resampled to 10 Hz.

A Morlet wavelet was used to produce time–frequency distributions for the signal pair SBP → EMGimp (muscle-pump baroreflex) and SBP → RR (cardiac baroreflex) (Garg et al., 2013; Garg et al., 2014). Monte-Carlo simulation was used to determine the significant coherence threshold (Xu et al., 2017). In this research, the muscle-pump baroreflex was investigated in a low-frequency band (LF, 0.07–0.15 Hz) previously linked to cardio-postural coupling and the muscle-pump baroreflex (Xu et al., 2017). The vagal cardiac baroreflex (Blaber et al., 2022) was investigated in the high-frequency band (HF, 0.15–0.5 Hz). The area above the significant coherence threshold in each frequency band was divided by the overall area of that frequency band to calculate the portion of the total time with active interaction (Fraction Time Active: FTA). The cross wavelet transform of the two signals was used to obtain the response gain value (Grinsted et al., 2004) and averaged over sections of significant WTC within each frequency range. The effectiveness of each interaction was further described using “Active Gain”, (Gain × FTA) (Xu et al., 2020).

The convergent cross-mapping technique was used to calculate the causal relationship between the signal pairs (EMGimp and SBP) and (RR and SBP) (Sugihara et al., 2012). Details on the methods may be found in Verma et al. (Verma et al., 2017a) and Sugihara et al.'s supplementary material (Sugihara et al., 2012). A two-dimensional plot (Active Gain vs. Causality) was utilized to show the correlation between causality and activity as they relate to the muscle-pump baroreflex and HDBR.

### Statistical analysis

The interquartile range approach for detecting outliers was adopted to ensure that all cardio-postural values and interrelationship factors of BP and muscle activity were meaningful throughout the preprocessing stage. If a value was 1.5 times the interquartile range, larger than the third quartile, or less than the first quartile, it was termed an outlier. The *winsorization* approach to treating outliers was independently applied to each of the four participant groups (Kwak and Kim, 2017).

Given the small numbers of participants in each group (*n* = 4–6) from males and females who were randomly assigned to two interventions (control and exercise), where not all response variables were normally distributed, we used a nonparametric ANOVA-type statistic (nparLD, F2-LD-F1 design) suggested by Brunner et al. (Brunner et al., 2002). The F2-LD-F1 design refers to an experimental design with two between-subjects factors (sex and intervention) and one within-subjects factor (test days). This design was employed to study the effect of sex, intervention, and test days as well as their interaction on the calculated response variables. To investigate the pairwise differences between BDC-1, R+0, and R+6 (time main effect), we applied multiple comparisons (LD-F1 design) with Bonferroni adjustment. Kruskal–Wallis test followed by Conover-Iman post-hoc test was used to study the differences between male controls, female controls, male exercise, and female exercise (treatment main effects) during BDC-1, R+0, and R+6. All statistical tests were performed using R (Team, 2011), and data are reported as significant (*p* < 0.05) or trends (0.1 > *p*≥ 0.05).

## Results

### Participants

Following the screening of volunteers with inclusion and exclusion criteria (Supplementary Material), twenty-three participants entered the study. These participants were randomized by the RI-MUHC staff into the four test groups and then into four campaign cohorts: one cohort of five and three cohorts of six individuals. One participant withdrew from the study during the head-down tilt portion, and two others developed medical conditions unrelated to bedrest, during the recovery phase and were removed from the study before completion. Therefore, data from twenty healthy men and women between 55–65 years of age were analysed (age: 58.7 ± 0.5 years, height: 1.67 ± 0.02 m, body mass: 70.2 ± 3.2 kg; mean ± SEM). The final group sizes were as follows: male controls (*n* = 5), female controls (*n* = 6), male exercise (*n* = 5), and female exercise (*n* = 4). All participants spent a total of 26 days (5 days of adaptation to the facilities, followed by 14 days of traditional six degrees of downward inclination bedrest in which participants used a pillow, and 7 days of recovery) at the Research Institute of the McGill University Medical Centre (RI-MUHC).

### Presyncope

Seven of the twenty participants were unable to complete the StS test on R+0 (Table 2). Six of the seven non-finishers were female. The male non-finisher was in the exercise group and was 39 s from completing the total 6-min stand. The female non-finishers were evenly split between the exercise and control groups; however, participants in the exercise group had the shortest times to presyncope of all non-finisher participants. These three participants all had less than the standardized analysis window of 180 s for WTC and causality analysis. One participant with 83 s was removed and the other two were analysed using a 140 s window (Table 2) reducing the analysis sample size for the female exercise group on R+0 to five.

**TABLE 2:** Bedrest exercise protocols. A combination of up to three per day were performed with a maximum total time of 62 min per day.

Sex	Intervention	Presyncope	Reason for termination	Total stand time (s)	Data analysis segment (s)
Female	Control	Yes	sudden ↓BP	269	180
		Yes	sweating, participant request	250	180
		Yes	sudden ↓BP	209	180
		No	------	360	180
	Exercise	Yes	dizziness, sudden ↓BP	83	X
		No	------	360	180
		No	------	360	180
		Yes	sudden ↓BP	151	140
		Yes	sudden ↓BP	145	140
		No	------	360	180
Male	Control	No	------	360	180
		No	------	360	180
		No	------	360	180
		No	------	360	180
		No	------	360	180
	Exercise	Yes	sudden ↓BP	321	180
		No	------	360	180
		No	------	360	180
		No	------	360	180
		No	------	360	180

### Cardiovascular and electromyography responses

**FIGURE 2:**
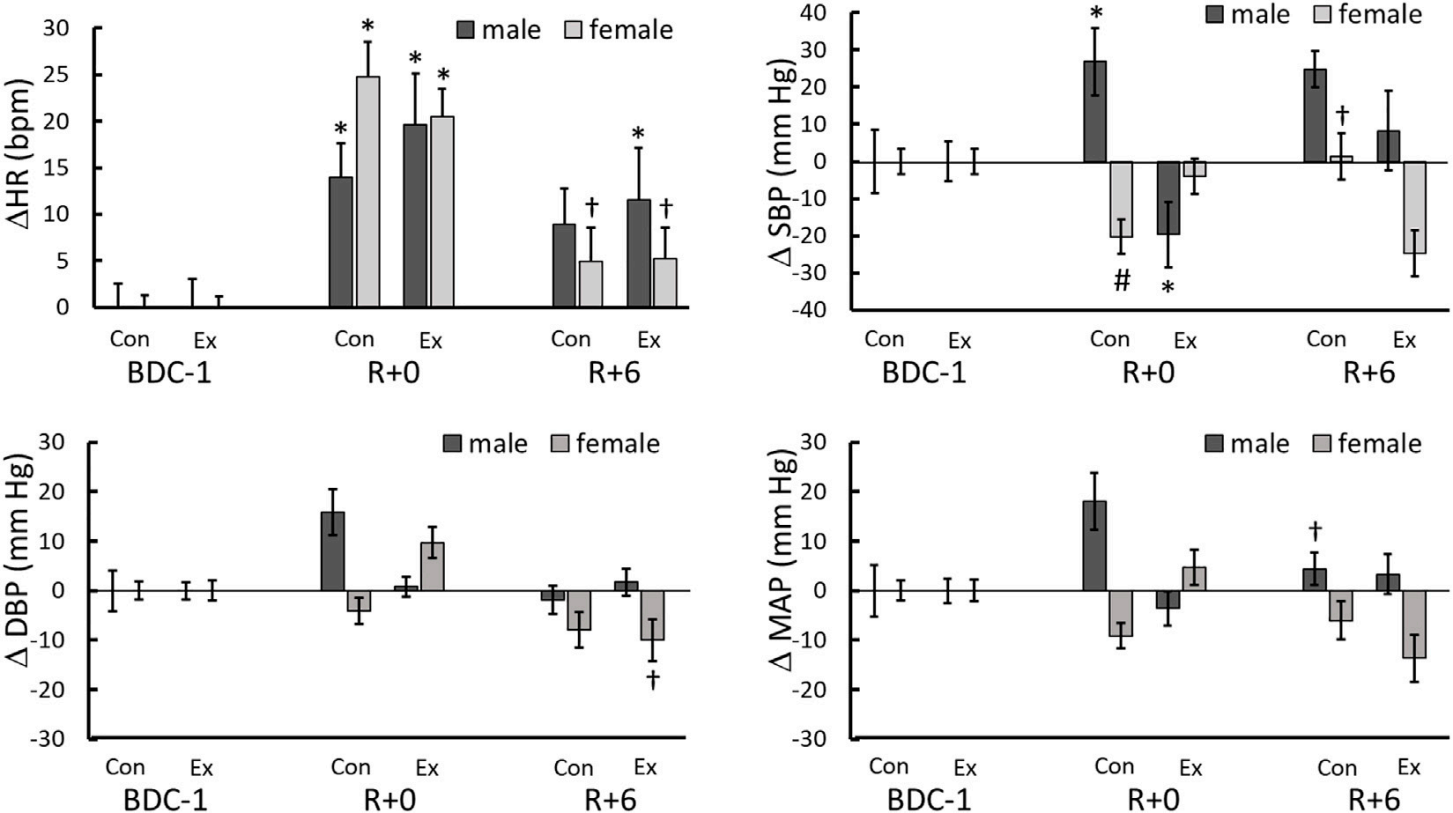
Heart rate and blood pressure changes from BDC-1 (increase: positive; decrease: negative) for different sex and intervention groups on R+0 and R+6. *: significantly different from BDC-1, †: R+6 different from R+0. #: different from males in same day and intervention. ‡: the control and exercise groups were significantly different for the same sex.

**FIGURE 3:**
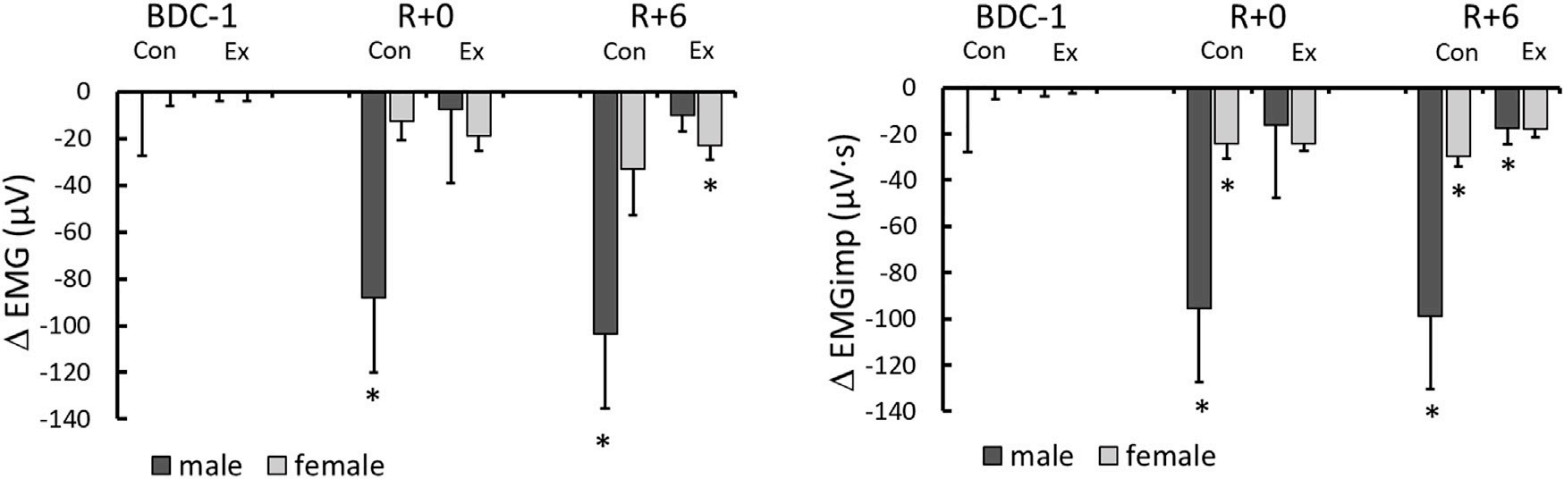
Electromyography (EMG) and electromygraphy impulse (EMGimp) changes from BDC-1 (increase: positive; decrease: negative) for different sex and intervention groups on R+0 and R+6. *: significantly different from BDC-1.

The cardiovascular and EMG measurements were influenced considerably by 14-days HDBR. Given the small sample size per group, differences were found in the baseline values. To examine post-bedrest responses, we first compared values in each group to their baselines (Table 3), then responses between groups were compared using changes in values from BDC-1; increases being positive and decreases being negative (Figures 2, 3).

**TABLE 3:** Mean (± standard error) standing cardio-postural values for different groups including male control group, male exercise group, female control group, and female exercise group on BDC -1 and R+0. Mean cardio-postural values were obtained from the stand phase of the supine-to-stand test. BDC -1: baseline data collection day −1; R+0: 2 h after the end of bedrest; R+6: 6 days after bedrest; HR: heart rate; SBP: systolic blood pressure; DBP: diastolic blood pressure; MAP: mean arterial pressure; EMG: electromyogram; EMGimp: Electromyogram beat-to-beat impulse.

Variable	Sex	Pre-bedrest (BDC -1)		Post bedrest (R+0)		Post bedrest (R+6)	
		Control	Exercise	Control	Exercise	Control	Exercise
HR (bpm)	Male	77.8 ± 2.5	71.5 ± 3.0	91.8 ± 2.6 *	91.1 ± 4.6 *	86.7 ± 2.8	83.0 ± 3.2 *
	Female	74.2 ± 1.3	83.3 ± 1.2	99.0 ± 3.5 *	103.8 ± 2.7 *	79.1 ± 1.0 †	88.5 ± 2.0
SBP (mmHg)	Male	126.7 ± 8.6	141.6 ± 5.3	153.6 ± 2.7 #	121.9 ± 7.0 *	151.5 ± 4.1	149.8 ± 8.1
	Female	117.9 ± 3.5	147.8 ± 3.5	97.6 ± 3.0 #‡	143.8 ± 3.2 ‡	119.33 ± 5.4	123.1 ± 5.3
DBP (mmHg)	Male	66.1 ± 4.1	65.3 ± 1.7	81.9 ± 2.3 ‡	66.1 ± 1.0 ‡	64.2 ± 1.7 †	67.0 ± 2.5
	Female	63.3 ± 1.8	76.5 ± 2.0	59.2 ± 1.9 ‡	86.2 ± 2.4 ‡#	55.4 ± 3.1	66.5 ± 3.4
MAP (mmHg)	Male	81.7 ± 5.2	83.7 ± 2.5	99.8 ± 2.4 #	80.1 ± 2.5	86.1 ± 2.3	84.8 ± 3.1
	Female	80.9 ± 2.0	96.96 ± 2.2	71.8 ± 1.7 *#‡	101.7 ± 2.8 ‡	74.9 ± 3.4	83.3 ± 3.9
EMG (µV)	Male	193.5 ± 27.6 #‡	73.0 ± 3.9 ‡	105.2 ± 2.6 *	65.6 ± 6.4	89.8 ± 3.7 #*	63.0 ± 3.0
	Female	86.7 ± 6.3 #	92.9 ± 3.9	74.2 ± 5.4	74.2 ± 5.3	53.8 ± 3.5 #	69.98 ± 3.4 *
EMGimp (µV·s)	Male	162.7 ± 27.8 ‡	65.0 ± 5.6 ‡	67.0 ± 2.6 *	48.8 ± 7.4	63.9 ± 4.0 *	47.5 ± 3.4 *
	Female	70.3 ± 4.8	66.6 ± 2.5	46.1 ± 4.1 *	42.6 ± 2.5 *	40.6 ± 2.1 *	48.7 ± 2.6

Legend: *: significantly different from BDC-1, #: significant difference between male and female participants in the same intervention group., ‡: on each day, the control and exercise intervention groups were significantly different for the same sex. Significance was set at *p* < 0.05.

During the quiet stand of the R+0 StS test, 2 h after the end of bedrest, a significant increase from the BDC-1 baseline in the average HR was observed in all study groups (*p* < 0.0001) (Table 3; Figure 2), with a significant reduction towards baseline values on R+6 in female (*p* = 0.019) but not the male participants (Figure 2).

The response of standing SBP, DBP, and MAP differed between the intervention and sex groups throughout test days. On R+0 the male control group had an increase in systolic blood pressure while the female control (*p* = 0.056) and male exercise (*p* = 0.068) groups trended in the opposite direction (Table 3; Figure 2). No change from baseline was observed with the female exercise group on R+0 or any group on R+6 (Table 3); however, significant reversals from R+0 occurred with the female control and male exercise SBP responses to standing. (Figure 2). No changes from baseline were found for DBP or MAP for all groups studied (Table 3); however, similar to SBP, there were trend reversals from R+0 to R+6 (Figure 2).

Overall lower leg muscle activity was only significantly reduced with HDBR in the male control group (Table 3). However, when EMG was integrated beat-to-beat (EMGimp), the effect was more dramatic in both male and female control groups, with more than a 33 and 25% reduction, respectively, from baseline on R+0. These changes persisted on R+6 at similar magnitudes (Figure 3).

### Muscle-pump baroreflex

Following HDBR, the skeletal muscle-pump’s ability to react to variations in BP was significantly reduced (Table 4). The FTA response varied across intervention and sex groups throughout the test days. Male exercise and female control groups had a substantial reduction in FTA on R+0 compared to pre-bedrest values (BDC-1), while no changes from baseline were found for other groups. Only the male exercise group increased significantly on R+6 (Table 4). With respect to the muscle-pump baroreflex, where skeletal muscle responds to changes in BP, only the male control group showed a significant reduction in SBP→EMG gain on R+0 from baseline. Although not significantly reduced on R+0, the male exercise group was significantly higher on R+6 than R+0 and not different from baseline (*p* = 0.006). No change over HDBR or recovery was observed in female participants.

**TABLE 4:** Wavelet transform analysis and convergent cross-mapping of systolic blood pressure and calf muscle electromyography impulse interactions during standing for different groups including male control group, male exercise group, female control group, and female exercise group on BDC -1 and R+0. BDC -1: baseline data collection day −1; R+0: 2 h after the end of bedrest; R+6: 6 days after bedrest; Gain: wavelet transform gain; FTA: fraction time active (above significant coherence threshold); causality: control directionality; LF: low frequency. Values are means (± standard error).

Variable	Sex	Pre-bedrest (BDC -1)		Post bedrest (R+0)		Post bedrest (R+6)	
		Control	Exercise	Control	Exercise	Control	Exercise
FTA (LF)	Male	0.30 ± 0.05	0.37 ± 0.03	0.22 ± 0.02	0.13 ± 0.01 *	0.19 ± 0.07	0.21 ± 0.01 †
	Female	0.35 ± 0.07	0.21 ± 0.02	0.25 ± 0.08 *	0.12 ± 0.02	0.35 ± 0.10	0.12 ± 0.02
Gain (LF) (μV⋅s/mmHg)	Male	0.71 ± 0.07	0.95 ± 0.10	0.45 ± 0.04*	0.56 ± 0.03	0.68 ± 0.07	0.78 ± 0.07 †
	Female	0.71 ± 0.07	0.51 ± 0.03	0.76 ± 0.08	0.64 ± 0.08	0.62 ± 0.12	0.66 ± 0.12
Causality (SBP → EMGimp)	Male	0.85 ± 0.01	0.87 ± 0.01	0.73 ± 0.02*	0.81 ± 0.02	0.80 ± 0.02	0.77 ± 0.02*
	Female	0.87 ± 0.02	0.84 ± 0.02	0.80 ± 0.03	0.81 ± 0.03	0.87 ± 0.01	0.80 ± 0.02
Causality (EMGimp → SBP)	Male	0.90 ± 0.01	0.93 ± 0.01	0.91 ± 0.01	0.92 ± 0.01	0.88 ± 0.02	0.91 ± 0.01
	Female	0.93 ± 0.01	0.91 ± 0.01	0.90 ± 0.02	0.92 ± 0.01	0.9 ± 0.01	0.85 ± 0.01

Legend: *: significantly different from BDC-1, †: R+6 different from R+0.

### Cardiac baroreflex

Our data from the coupling of blood pressure and heart rate (SBP→RR) showed that the cardiovascular baroreflex was affected by HDBR (Table 5). The fraction that the cardiac baroreflex was active (FTA) was significantly decreased in females only after bedrest (R+0), but this recovered to baseline levels by R+6. The exercise intervention had no discernible effect on the outcomes as cardiac baroreflex gain was significantly reduced following bedrest on R+0 in all groups studied. The male exercise group had the greatest reduction in cardiac gain (∼65% on R+0), and both male groups remained depressed on R+6, while both female groups had returned to baseline (Table 5).

**TABLE 5:** Wavelet transform analysis and convergent cross-mapping of systolic blood pressure and cardiac arterial interactions during standing for different groups including male control group, male exercise group, female control group, and female exercise group on BDC -1 and R+0. BDC -1: baseline data collection day −1; R+0: 2 h after the end of bedrest; R+6: 6 days after bedrest; SBP→RR: Neural cardiac baroreflex direction; RR→SBP: mechanical non-baroreflex direction; Gain: wavelet transform gain; FTA: fraction time active (above significant coherence threshold); causality: control directionality; HF: high frequency. Values are means (± standard error).

Variable	Sex	Pre-bedrest (BDC -1)		Post bedrest (R+0)		Post bedrest (R+6)	
		Control	Exercise	Control	Exercise	Control	Exercise
FTA (HF)	Male	0.46 ± 0.03	0.38 ± 0.04	0.35 ± 0.04	0.26 ± 0.02	0.39 ± 0.02	0.39 ± 0.01
	Female	0.36 ± 0.07	0.47 ± 0.06	0.22 ± 0.07 *	0.30 ± 0.06 *	0.42 ± 0.07	0.45 ± 0.06
Gain (HF) (ms/mmHg)	Male	5.09 ± 0.39	10.85 ± 1.25	2.56 ± 0.37 *	3.32 ± 0.30 *	3.03 ± 0.40 *	3.16 ± 0.17 *
	Female	9.43 ± 1.32	5.25 ± 1.10	3.13 ± 0.61 *	2.09 ± 0.30 *	5.63 ± 0.36	4.12 ± 0.64
Causality (SBP → RR)	Male	0.95 ± 0.01	0.95 ± 0.01	0.93 ± 0.01	0.91 ± 0.01	0.93 ± 0.01	0.92 ± 0.01
	Female	0.90 ± 0.017	0.88 ± 0.01	0.95 ± 0.01 *	0.88 ± 0.03	0.88 ± 0.03	0.89 ± 0.01
Causality (RR → SBP)	Male	0.92 ± 0.01	0.95 ± 0.01	0.93 ± 0.01	0.87 ± 0.03	0.93 ± 0.01	0.94 ± 0.01
	Female	0.94 ± 0.01	0.89 ± 0.01	0.91 ± 0.01	0.94 ± 0.01	0.91 ± 0.01	0.85 ± 0.01

Legend: * significantly different from BDC-1.

### Causality

Significant changes in SBP→EMGimp causality were only seen in the male study participants. On R+0, CCM analysis of SBP→EMGimp directional coupling (baroreflex) revealed a substantial reduction in causality in the male control group (*p* < 0.0001), which recovered by R+6. This reflex muscle-pump baroreflex causality trended lower in the male exercise group on R+0 (*p* = 0.07), but by R+6 this became significantly reduced from baseline (Table 4). In the opposite (muscle-pump mechanics) direction (EMGimp→SBP), there was no change in causality, with a value that remained constant at a mean value of 0.91 ± 0.01.

Causality for the female control group post HDBR cardiac baroreflex (SBP→RR) increased but returned to baseline by R+6. There was no change in male causality related to HDBR. There was also no change in the causal effect of heart rate on blood pressure (RR→SBP, cardiac mechanics) in any group.

### Active gain vs. causality

**FIGURE 4:**
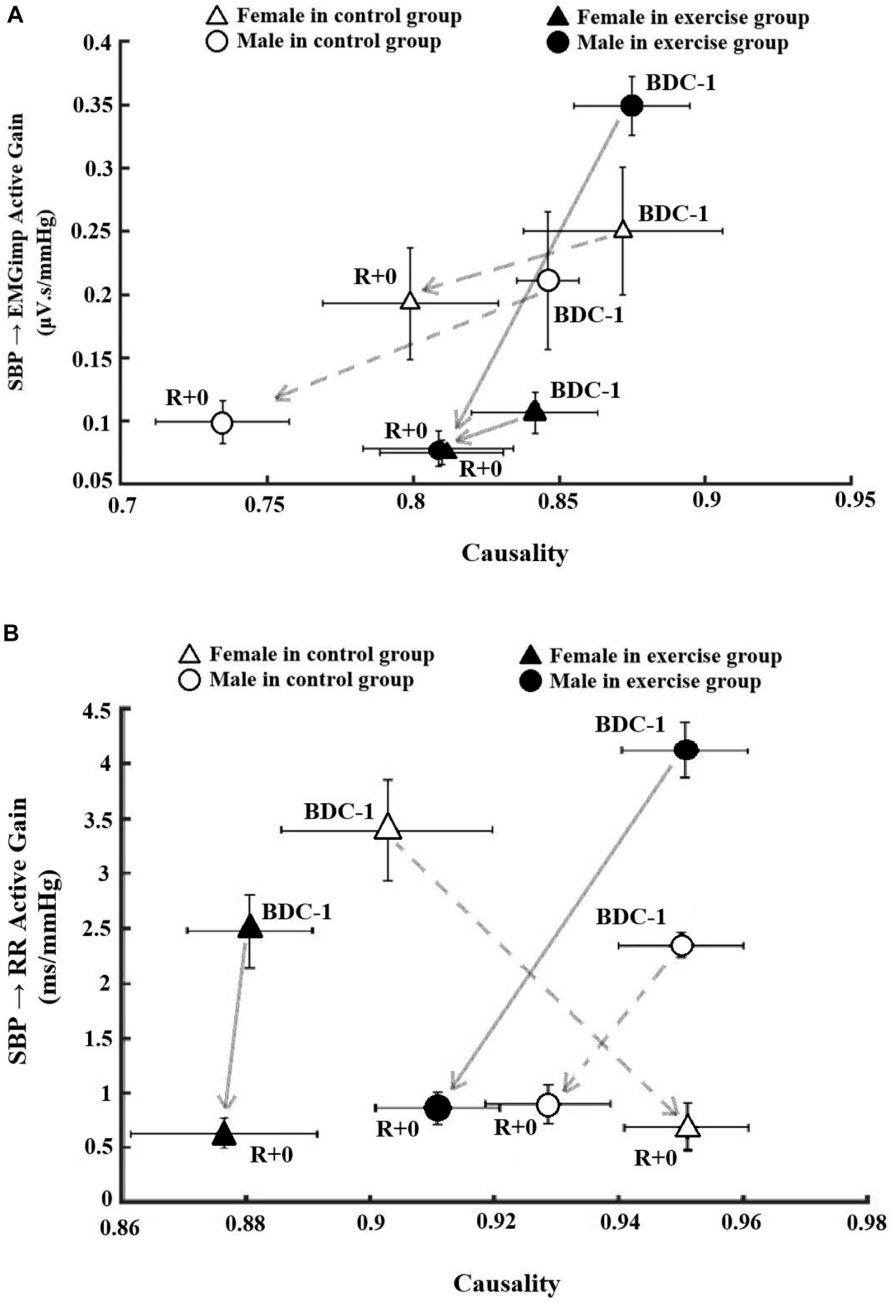
The association between causality and low frequency Active gain as a function of active interaction time (Active Gain: Gain X fraction time active) on pre bedrest (BDC-1) and R+0 related to **(A)** skeletal muscle-pump baroreflex system and **(B)** cardiac baroreflex system. The data in the circles are associated with male participants, while the data in the triangles are related to female participants. Filled markers indicate the exercise groups in both sexes.

To compare pre- and post-bedrest baroreflex responses, muscle-pump and cardiac baroreflex active gain, which is the product of gain and FTA (Gain × FTA), were plotted as a function of causality on BDC-1 and R+0 for all groups (Figure 4). There were different reactions in terms of baroreflex functionality between the intervention and sex groups pre- and post-bedrest. Regarding the muscle-pump baroreflex, when compared to BDC-1, the male exercise group had the greatest reduction in muscle-pump active gain, while the male control group had the largest decrease in causality (Figure 4A). Females in both the control and exercise groups had more mild results than males in the same group in terms of muscle-pump interactions (Figure 4A).

The intervention and sex groups’ responses in cardiac baroreflex functionality were more dramatic following HDBR compared to muscle-pump baroreflex outcomes (Figure 4B). Although all groups studied had substantial decreases in cardiac baroreflex active gain, the only significant reduction was found in the female exercise group. The male exercise group, on the other hand, had both active gain and causality reductions following HDBR that were larger than the male control group. The female control group was the only group that exhibited a reversed direction of stronger causality and reduced active gain on R+0 compared to baseline on BDC-1 (Figure 4B).

## Discussion

Our new findings from the Canadian aging and inactivity study highlight the detrimental effects of bedrest on homeostatic mechanisms responsible for functional daily ambulatory activities. This was particularly serious with the female participants of whom 60%, compared to 10% of male participants, were unable to complete 6 minutes of stand just hours after exiting from 14 days of bedrest. Given the drastic consequences on orthostatic tolerance, this paper is focused on two major components of the blood pressure control system, the cardiac and muscle-pump baroreflexes. Our results into orthostatic reflexes reveal that following 2 weeks of bedrest, skeletal muscle activation and heart rate changes in connection to BP regulation were reduced in older participants. To our knowledge, this is the first study to report changes in cardio-postural interactions in both sexes and older persons after extended bedrest confinement.

The findings are particularly relevant for understanding orthostatic intolerance (OI), a syndrome that affects both older (Goswami, 2017) and younger (Xu et al., 2020) people after bedrest, simulated microgravity (e.g. HDBR) (Goswami et al., 2017), or astronauts after spaceflight (Blaber et al., 2011; Blaber et al., 2013; Blaber et al., 2022), respectively. Furthermore, the older composition of participants in this study adds to our understanding of the interaction of age with inactivity on the cardio-postural control system.

### Muscle-pump baroreflex

We recently provided evidence of the importance of lower limb muscular contractions for the maintenance of standing blood pressure (Xu et al., 2020). These contractions compress underlying veins, resulting in the pumping of venous blood pooled in the legs back to the heart (muscle-pump) in a coordinated response raising venous return to counteract reductions in BP (Verma et al., 2017a; Verma et al., 2019). As a result, BP management during standing necessitates input from the cardiovascular, postural, and musculoskeletal systems. We also showed that this blood pressure related muscle-pump reflex was impaired in middle-aged male participants following 60 days of bedrest inactivity (no exercise intervention) (Xu et al., 2020). This is the first study where muscle-pump baroreflex has been investigated in women following bedrest.

We used EMG impulse as an indicator of the beat-to-beat translation of muscle activity (EMG) to the cardiovascular system via the skeletal muscle-pump. Given the considerable variation in baseline EMG and EMGimp across groups (Table 1)—most likely related to the small sample sizes—changes from baseline were used to assess intergroup effects. EMGimp decreased significantly only in the male and female control groups on R+0 and R+6 compared to pre-HDBR values (Figure 3). Major declines in EMGimp were not observed in the exercise groups, although the male exercise group showed a significant decline on R+6, this value was not different from R+0, which had much higher variation, and not different from the female exercise group. Of the two sexes, males had the largest decreases in EMGimp. Males are predicted to have more muscular deconditioning and dramatic alterations since they have larger muscle mass than females. Smaller, yet consistent declines in EMGimp were also seen in the female control group. In contrast, no change in EMGimp from baseline was seen in male or female exercise participants (Figure 3), indicating that they were better able to sustain muscle-pump capacity after lengthy periods of bedrest. These data are a clear indication that the skeletal muscle’s capacity to pump blood back to the venous circulation in response to BP variations was impaired by 14 days of bedrest but was preserved by the daily exercise regime.

Our previous research with healthy younger males found that along with reduced EMGimp there was a changed relationship of BP to muscular activation (gain, FTA, causality) after bedrest and inactivity which indicated not only a probable drop in reflex output to the muscle but also a variation in activation (Xu et al., 2020). In this study, we examined the variations among the four studied groups to see how biological sex and exercise intervention affected muscular activation through the muscle-pump baroreflex. Following HDBR, there was a considerable decrease in the percentage of significant coherence over the duration of the stand, as expressed through FTA, in the male exercise and female control groups. Given the small sample size per group and mixed results, these data implied that after an extended period of immobility, the prescribed exercises may have a lesser impact on preserving FTA in male participants, and improving the training parameters such as loading frequency, workload, rate, and rest period should be studied more closely.

Like EMGimp, muscle-pump (SBP → EMG) gain was reduced the greatest in the male control group; however, unlike EMGimp no reduction was observed in female controls (Table 4). In fact, there was an across-the-board retention of muscle-pump baroreflex gain in all female participants post-bedrest. The male exercise muscle-pump baroreflex gain on R+0 was not decreased significantly but showed a significant increase from R+0 on the last day of measurement (R+6) which may indicate a positive latent effect of exercise in the male participants (Table 4).

Causality, a measure of the strength of coupling between signals was reduced in the muscle-pump baroreflex direction (SBP → EMGimp) on R+0 and R+6 in the male control group only. This supports our previously reported reduction in coupling between blood pressure and the skeletal muscle-pump following bedrest in male participants (Xu et al., 2020). This decline in muscle-pump directional influence was not observed in the male exercise group or in the female participants. These data further solidify the beneficial effects of exercise in older males. No change in muscle-pump baroreflex causality in the female participants was observed, suggesting a possible sex-related differential effect of bedrest which was also observed in muscle-pump baroreflex gain. However, caution must be taken in interpretation given the small sample size and moderately short time in bedrest.

The absence of causality changes in the inverse direction (EMGimp → SBP) implies that HDBR did not affect the mechanical connection between muscle-pump activity and BP. These data add further support to our hypothesis that variations in BP control are reflex/neurally mediated rather than caused by changes in muscle-pump mechanics (Xu et al., 2020). Finally, we examined the interaction between muscle-pump activity, the product of gain and FTA, with causality. Figure 4A showed that regardless of baseline active gain, exercise limited the reduction in EMG to BP coupling, as shown by the greater changes in causality in the control groups.

### Cardiac baroreflex

Another critical component of the autonomic response to sudden reductions in blood pressure upon standing, is the cardiac baroreflex. Efferent neural pathways increase heart rate, systemic vascular resistance, and cardiac contractility via vagal withdrawal and sympathetic activation. Reductions in cardiac arterial baroreflex response have long been recorded for both short (Fritsch-Yelle et al., 1994; Gisolf et al., 2005; Verheyden et al., 2007) and long-term (Hughson et al., 2012) spaceflight. Bedrest has also been linked to decreased arterial baroreflex (Convertino et al., 1990; Traon et al., 1997; Iwasaki et al., 2000; Hirayanagi et al., 2004).

We observed elevated standing HR following HDBR (Table 3; Figure 2), an indication of greater vagal withdrawal and cardiovascular deconditioning, which continued until R+6. This increase was global, indicating that post-bedrest neither the biological sex of the participant nor exercise impacted the outcome. Raised HR upon standing is related to reduced central blood pressure, with greater HR increases commonly observed after bedrest as a compensatory reaction to increased venous pooling in the lower limbs through a lack of enhanced vasoconstriction (Feldstein and Weder, 2012; Veronese et al., 2015; Möstl et al., 2021). Similar to our discussion of EMG, changes in SBP from baseline were used to assess intergroup effects. Post-bedrest, in response to standing, male control participants had elevated SBP whereas the female control and male exercise participants had lower SBP (Figure 2). While, neither DBP nor MAP was altered significantly from baseline across test days in any test groups it must be noted that these values were averaged prior to presyncope. Blood pressure is protected at all costs and is not a reliable early predictor of presyncope (Buszko et al., 2019). Not until cardiovascular decompensation occurs will blood pressure decrease. Elevated SBP, possibly due to greater vasoconstriction in the male participants, may partially explain the significantly lower number of presyncopal males since cerebral perfusion may have been better protected than in female participants (Buszko et al., 2019).

Our data revealed a considerable reduction in cardiac baroreflex gain after HDBR (R+0) in all studied groups as well as cardiac baroreflex FTA. These data do not support the hypothesis that the prescribed exercises during bedrest would maintain cardiac baroreflex in older persons, although the SBP data is suggestive of protective vascular effects of exercise in the male participants. Differences between the male and female participants suggest that along with vascular control there are unique sex-related cardiac baroreflex control adaptations to exercise with bedrest deconditioning.

Unlike their male counterparts, female participants had significantly reduced FTA on R+0. This is an indication that on R+0 baroreflex mediated autonomic signals to the heart were either less frequent of shorter in duration compared to pre-bedrest and to males. Furthermore, the female control participants had a significant increase in causality on R+0, where all other groups, including the female exercise group, had no notable change in causality. When gain and FTA were combined as active gain and plotted with causality this contrast was more evident (Figure 4B). While male control and male and female exercise groups showed parallel declines in active gain and causality from pre-to post-bedrest, the female control group had an increase in causality while exhibiting a similar drop in active gain. Although the number of participants was four, we can postulate on a mechanism. The female control participants had significantly lower SBP than pre-bedrest and the lowest SBP of all the groups (Table 1). This may have led to an increase in cardiac causality as compensation for BP dysregulation during standing.

We expected similar losses in the cardiac arterial baroreflex after comparable durations (∼2 weeks) of inactivity from HDBR or after spaceflight. Blaber et al. (Blaber et al., 2022) presented spaceflight data of equivalent duration (8–16 days) to this bedrest study using similar analyses on 10-min stand tests pre- and post-spaceflight. The astronauts did not have a significant decrease in baroreflex gain on landing day but did have a similar significant decrease in FTA. The astronauts also had a significant decrease in causality, not seen in our participants, with the women in our control group exhibiting and increase in causality. Some of the differences that may have contributed to dissimilarity could be: 1) weightlessness and HDBR not being equal in terms of unloading of the body since HDBR only removes the gravitational gradient from the head-foot axis of the body; 2) the astronauts had a mean age was 39 ± 5 years, 20 years less than that of our participants; 4) the astronauts would have physically and mentally trained for weightless for several years prior to flight while our participants, although fit, as defined by the inclusion criteria (Supplementary Material), may have had only months to prepare for HDBR; and 5) it is likely that the astronauts may not have had opportunity to exercise as extensively as our HDBR participants in the exercise group and any biological sex-related interactions with exercise would not have been observed. Further research is needed to determine the impact of immobilization/spaceflight length, effects of biological sex, and different exercise regimes on the degree of cardiac baroreflex impairment.

### Reflections on space-based exercises as countermeasures during HDBR in older persons

Flight regulations on the International Space Station mandate that all crew members on long-duration missions perform exercise, which now makes it impossible to study the consequences of no exercise on the physiological impacts of spaceflight. As a result, comparison to earlier missions (Sibonga et al., 2015) or to a period before a substantial change in hardware (English et al., 2015; Sibonga et al., 2015), such as the replacement of iRED with ARED, is the only approach to assess the efficacy of current exercise countermeasures in space. The restricted opportunity to conduct controlled intervention studies, both in space and in spaceflight analogues such as HDBR, is a substantial hurdle to developing a new exercise countermeasure (Traon et al., 2007; Hargens and Vico, 2016). An exercise training intervention study is expensive and time-consuming in space. The “SPRINT” research (Rice, 2019) conducted by NASA was a unique case of a supervised, in-flight study to assess the efficacy of high intensity, low volume exercise training regimen, which demonstrated promising results in both HDBR (Ploutz-Snyder et al., 2018) and microgravity (Goetchius et al., 2020). Even in this case, the control group was not fully deprived of physical activity and continued to perform routine ISS countermeasure exercises.

Terrestrial investigations (e.g., HDBR campaigns) are likewise expensive and complicated, albeit not as much as space research, but they provide more experimental control and allow hypotheses to be addressed more rapidly. Ground-based studies of wider scope (i.e., there is no restriction on time, frequency, or intensity/overload) has allowed for the widespread acceptance of terrestrial exercise training ideas such as continuous and interval-type aerobic exercise and high-intensity, multi-set/rep resistance training And the improvement of ISS exercise countermeasure hardware (Scott et al., 2019). The combination of aerobic, HIIT, and resistance exercises employed in this study was only partially successful in preserving the muscle-pump baroreflex even though there was significant preservation of beat-to-beat muscle activity during standing. However, given the reductions in active time and reflex causality seen in some groups, the preservation of beat-to-beat muscle activity may not have been as effective with counteracting the reduction in reflex activity and causal coupling of blood pressure to muscle contractions and heart rate changes. Given the small sample size per group, our findings may suggest that the benefits of exercise intervention differed by biological sex and that they might be if tailored to biological sex. Furthermore, the exercises prescribed in this study were ineffectual in preserving cardiac baroreflex function and additional research must be conducted to assess the interrelationship between the combinations of exercises and components of the baroreflex system while taking into consideration sex-specific physiological effects.

Only menopausal women were eligible for participation in this study. Due to ethical concerns of severe detrimental outcomes of bedrest in elderly persons, the Canadian aging and inactivity study’s participant age range was 55–65. This was to limit acute and long-term impacts on health yet provide sufficient data to expand our knowledge of the effects of inactivity on the elderly. One of the most important factors associated with cardiovascular disease in both men and women is the stiffening of the arterial structure that occurs as we age. However, a sudden drop in oestrogen levels in the bloodstream could contribute to an increase in blood pressure through mechanisms that are still not fully understood, such as a direct effect on the arterial wall, activation of the renin-angiotensin system and the sympathetic nervous system. (Izumi et al., 2007; Taddei, 2009; Tikhonoff et al., 2019). All elderly women are menopausal, however not all females are menopausal in our study’s age range. If we were to include both perimenopausal and menopausal women, we would have been unable to accurately compare the women in the two groups (Taddei, 2009; Tikhonoff et al., 2019); therefore, it was important to exclude women who were not menopausal. Furthermore, if we were to include perimenopausal and menopausal women, we would be unable to accurately draw conclusions based on a mix of peri- and menopausal women in the control and exercise groups (Taddei, 2009; Tikhonoff et al., 2019), and it would make it difficult to draw comparisons with the outcomes of young females who are typically involved in bed rest studies.

### Presyncope

Despite declines in both muscle-pump and cardiac baroreflexes, the male participants in our study had better outcomes related to presyncope compared to the female participants. Given that the prescribed exercises (Hedge et al., 2022) in HDBR were not an effective countermeasure for preserving the cardiac baroreflex an overall comparison between the two study samples is justified. Although not the only outcome expected from the implementation of exercise, prevention of syncopal events is a high priority with hospitalized older patients as this can lead to falls, co-morbidities, and death. From a space health perspective, loss of orthostatic tolerance can have operational consequences if astronauts cannot perform mission tasks within hours or days of landing on a planetary body.

In this regard, we can look at the data from shuttle astronauts who were exposed to gravitational unloading for a similar number of days (Blaber et al., 2011; Blaber et al., 2022). The fraction of presyncopal men and women following spaceflight (2/19 men, 5/7 women) was the same as in the current bedrest study (1/10 men, 6/10 women). However different the environment experienced between the two types of participants; the physiological outcome (presyncope) was the same. Orthostatic intolerance post-spaceflight in this cohort of astronauts has been attributed to reduced adrenergic vasoconstrictor response (Fritsch-Yelle et al., 1994), impaired cerebral autoregulation (Blaber et al., 2011) and decreased cardiac baroreflex (Blaber et al., 2022). Although we did not assess cerebral autoregulation in this study, our data show decreased cardiac baroreflex and blood pressure differences between groups suggestive of reduced vasoconstrictor response. We also have additional results from the muscle-pump baroreflex which was not available from the astronauts.

To provide a better understanding of the mechanisms associated with presyncope in our participants, we reanalysed the data using presyncope—those who finished the stand test (finishers) and those who did not (non-finishers)—to delineate participants, rather than biological sex. Only one variable, SBP-EMG (muscle-pump baroreflex) causality had a presyncope-specific interaction with bedrest. Prior to bedrest both non-finishers’ and finishers’ muscle-pump baroreflex causality were not different (0.87 ± 0.03, 0.86 ± 0.03, respectively) (*p* = 0.998), however, on R+0 non-finishers’ causality remained the same (0.87 ± 0.03) while finishers’ causality was significantly lower (0.74 ± 0.03) (*p* = 0.045). Finally, on R+6, non-finishers (0.84 ± 0.03) and finishers (0.79 ± 0.03) were again not significantly different (*p* = 0.797).

These results may reveal a global underlying response to severe orthostatic stress that was not observed in our analyses due to sex related differences in physiology and susceptibility to post-HDBR orthostatic intolerance. In the analysis presented in the results, we focused on biological sex and the exercise intervention. As a result, the data associated with presyncope was spread over several groups, predominately female. None of the males in the control group was presyncopal and had a significantly lower causality (Table 4) than pre-bedrest. The lone presyncopal male was in the exercise group (*n* = 5) with an SBP-EMG causality of 0.95 which skewed the value higher. Similarly, the lone finisher in the female control group (*n* = 4) had a causality of 0.74. and the mean value for the three finishers in the exercise group (*n* = 6) was (0.71 ± 0.08).

The relatively large size of the non-finisher group compared to any of the prescribed groupings has provided a unique opportunity to explore the baroreflex mechanisms employed to prevent orthostatic hypotension and fainting. None of the variables associated with the cardiac or vascular components (Tables 1, 2) were found to distinguish between non-finishers and finishers. This would suggest that the functional contributions of these two branches of the baroreflex system were equally engaged to a similar extent during stand on any given day of measurement. Skeletal muscle contractions can enhance venous return through the pumping of blood up the veins in the leg through one-way valves. In this study we found that beat-to-beat EMG output to these muscles was reduced following bedrest (Table 1). Similarly, there were reductions in muscle-pump baroreflex FTA and gain which were blunted by exercise. However, none of these were found to be related with impending syncope indicating that the baroreflex system response, although operational, was limited in the scope to which these could be altered for preventing hypotension.

A greater muscle-pump baroreflex causality in the non-finisher group implies a tighter reflex coupling of blood pressure to skeletal muscle contractions. That is, changes in blood pressure are more closely translated to a change in muscle activity which may provide a more coordinated response to hypotension. That this was observed only in the non-finisher group could be evidence that this mechanism is one of final resort for a compromised cardiovascular system, that may have been sufficient for the finishers, but not the non-finishers. Inferential evidence for the existence of leg muscle activity being associated with hypotension and orthostatic tolerance comes from the observation of increased postural sway in persons who have orthostatic hypotension based on head-up tilt or lower body negative pressure test, but do not faint in stand tests (Claydon and Hainsworth, 2005). Astronauts, as we have examined earlier in this paper, are also susceptible to OH and have greater sway post-flight (Speers et al., 1998), while patients with autonomic failure often exhibit fidgeting leg behaviours when sitting (Cheshire, 2000).

### Limitations and future work

The participants in this study were selected from a healthy older population aged 55–66 years old; however, many older people are on several medications and have substantial sarcopenia even before being placed on bedrest (Blain et al., 2016; Goswami, 2017). They are frequently confined to bed owing to acute illnesses, severe injuries, procedures, or chronic ailments. Future research should investigate how different lengths of bedrest confinement affect cardio-postural connections in elderly people. This is significant because falls and fall-related injuries are frequently caused by a change in posture (upon standing from supine or sitting (Rapp et al., 2012; Goswami, 2017; Trozic et al., 2020). Future bedrest research should also include larger sample numbers in both biological sexes due to considerable sex-related variations and interindividual variability.

Physical exercise has been highlighted as a key strategy in reducing the negative consequences of bedrest confinement (Schneider et al., 2016; Ploutz-Snyder et al., 2018); however, more research is needed to compare distinct exercise types as modified and individualized exercise countermeasures for both sexes. Furthermore, sex-related differences in this study imply that the exercises should be designed specifically for each sex, which should be investigated further. More research also should be conducted to optimize training factors such as loading frequency, workload, pace, rest duration, and particular exercise “dosage” for each individual. Cognitive training (Goswami et al., 2015), LBNP (Goswami et al., 2019), pharmaceutical intervention (Lee et al., 2020), and artificial gravity (Evans et al., 2018) are further therapies that might be examined; all these have been shown to improve the symptoms of bedrest-induced physiological deconditioning. The findings can then be utilized to create and improve effective countermeasures.

Other factors that may alter postural responses, such as visual (eyes closed during testing) and vestibular inputs, were not included in the cardio-postural model presented in this article. In future investigations, a more comprehensive model combining the aforementioned factors should be adopted and examined.

## Conclusion

This study evaluated the effect of 14 days of 6-degree head-down tilt bedrest (HDBR) with or without combined lower body strength, aerobic, and high-intensity interval training (HIIT) exercise countermeasures on the muscle-pump baroreflex in older adults. Physical inactivity through bedrest reduced both cardiac and muscle-pump baroreflex activation (reduced gain and FTA) during a free-standing orthostatic challenge. The exercise intervention of upper and lower body strength, aerobic, and HIIT exercise countermeasures implemented in this first Canadian aging and inactivity study (CAIS) was not found to influence the decline in cardiac baroreflex and was only partially successful in preserving the muscle-pump baroreflex even though there was significant preservation of beat-to-beat muscle activity during standing. Further analysis into the interaction between muscle activation during exercise in relation to that during the blood pressure reflex is needed to expand our understanding of the neural coupling involved.

## Data Availability

The datasets presented in this article are not readily available because data may only be shared for the use under which it was ethically approved. Requests to access the datasets should be directed to andrew_blaber@sfu.ca.

## References

[B1] Antonutto G. Di Prampero P. (2003). Cardiovascular deconditioning in microgravity: Some possible countermeasures. Eur. J. Appl. Physiol. 90 (3), 283–291. 10.1007/s00421-003-0884-5 12851824

[B2] Blaber A. P. Goswami N. Bondar R. L. Kassam M. S. (2011). Impairment of cerebral blood flow regulation in astronauts with orthostatic intolerance after flight. Stroke 42 (7), 1844–1850. 10.1161/STROKEAHA.110.610576 21617145

[B3] Blaber A. P. Goswami N. Xu D. (2022). Prolonged unloading of the cardiovascular system during bedrest and spaceflight weakens neural coupling between blood pressure and heart rate. Acta Astronaut. 195, 567–573. 10.1016/j.actaastro.2022.03.009

[B4] Blaber A. P. Landrock C. K. Souvestre P. A. (2009). Cardio-postural deconditioning: A model for post-flight orthostatic intolerance. Respir. Physiol. Neurobiol. 169, S21–S25. 10.1016/j.resp.2009.04.007 19379846

[B5] Blaber A. P. Zuj K. A. Goswami N. (2013). Cerebrovascular autoregulation: Lessons learned from spaceflight research. Eur. J. Appl. Physiol. 113 (8), 1909–1917. 10.1007/s00421-012-2539-x 23132388

[B6] Blain H. Masud T. Dargent-Molina P. Martin F. Rosendahl E. Van Der Velde N. (2016). A comprehensive fracture prevention strategy in older adults: The European union geriatric medicine society (EUGMS) statement. Aging Clin. Exp. Res. 28 (4), 797–803. 10.1007/s40520-016-0588-4 27299902

[B7] Brunner E. Domhof S. Langer F. (2002). Nonparametric analysis of longitudinal data in factorial experiments. Wiley-Interscience, 1.

[B8] Buszko K. Kujawski S. Newton J. L. Zalewski P. (2019). Hemodynamic response to the head-up tilt test in patients with syncope as a predictor of the test outcome: A meta-analysis approach. Front. Physiol. 10, 184. 10.3389/fphys.2019.00184 30899228 PMC6416221

[B9] Cheshire W. P. (2000). Hypotensive akathisia: Autonomic failure associated with leg fidgeting while sitting. Neurology 55 (12), 1923–1926. 10.1212/wnl.55.12.1923 11134400

[B10] Chodzko-Zajko W. J. Proctor D. N. Singh M. A. F. Minson C. T. Nigg C. R. Salem G. J. (2009). Exercise and physical activity for older adults. Med. Sci. sports Exerc. 41 (7), 1510–1530. 10.1249/mss.0b013e3181a0c95c 19516148

[B11] Claydon V. E. Hainsworth R. (2005). Increased postural sway in control subjects with poor orthostatic tolerance. J. Am. Coll. Cardiol. 46 (7), 1309–1313. 10.1016/j.jacc.2005.07.011 16198849

[B12] Clément G. (2011). Fundamentals of space medicine. Springer science & business media. Springer, 1.

[B13] Convertino V. A. (1996). Exercise as a countermeasure for physiological adaptation to prolonged spaceflight. Med. Sci. Sports Exerc. 28 (8), 999–1014. 10.1097/00005768-199608000-00010 8871910

[B14] Convertino V. A. Doerr D. F. Eckberg D. L. Fritsch J. M. Vernikos-Danellis J. (1990). Head-down bed rest impairs vagal baroreflex responses and provokes orthostatic hypotension. J. Appl. Physiol. 68 (4), 1458–1464. 10.1152/jappl.1990.68.4.1458 2347788

[B15] Convertino V. A. Doerr D. F. Mathes K. Stein S. Buchanan P. (1989). Changes in volume, muscle compartment, and compliance of the lower extremities in man following 30 days of exposure to simulated microgravity. Aviat. Space Environ. Med. 60 (7), 653–658. 2764848

[B16] English K. L. Lee S. Loehr J. A. Ploutz–Snyder R. J. Ploutz–Snyder L. L. (2015). Isokinetic strength changes following long-duration spaceflight on the ISS. Aerosp. Med. Hum. Perform. 86 (12), A68–A77. 10.3357/AMHP.EC09.2015 26630197

[B17] Evans J. M. Knapp C. F. Goswami N. (2018). Artificial gravity as a countermeasure to the cardiovascular deconditioning of spaceflight: Gender perspectives. Front. Physiol. 9, 716. 10.3389/fphys.2018.00716 30034341 PMC6043777

[B18] Feldstein C. Weder A. B. (2012). Orthostatic hypotension: A common, serious and underrecognized problem in hospitalized patients. J. Am. Soc. Hypertens. 6 (1), 27–39. 10.1016/j.jash.2011.08.008 22099697

[B19] Fritsch-Yelle J. M. Charles J. B. Jones M. M. Beightol L. A. Eckberg D. L. (1994). Spaceflight alters autonomic regulation of arterial pressure in humans. J. Appl. Physiol. 77 (4), 1776–1783. 10.1152/jappl.1994.77.4.1776 7836199

[B20] Garg A. Xu D. Blaber A. P. (2013). Statistical validation of wavelet transform coherence method to assess the transfer of calf muscle activation to blood pressure during quiet standing. Biomed. Eng. Online 12 (1), 1–14. 10.1186/1475-925X-12-132 24365103 PMC3879179

[B21] Garg A. Xu D. Laurin A. Blaber A. P. (2014). Physiological interdependence of the cardiovascular and postural control systems under orthostatic stress. Am. J. Physiol. Heart Circ. Physiol. 307 (2), H259–H264. 10.1152/ajpheart.00171.2014 24858845

[B22] Gisolf J. Immink R. Van Lieshout J. Stok W. Karemaker J. (2005). Orthostatic blood pressure control before and after spaceflight, determined by time-domain baroreflex method. J. Appl. Physiol. 98 (5), 1682–1690. 10.1152/japplphysiol.01219.2004 15649869

[B23] Goetchius L. Scott J. English K. Buxton R. Downs M. Ryder J. (2020). “High intensity training during spaceflight: Results from the SPRINT study,” in Proceedings of the NASA human research program investigators’ workshop ‘human exploration and discovery: The moon, Mars and beyond), 22–25.

[B24] Goswami N. (2017). Falls and fall-prevention in older persons: Geriatrics meets spaceflight!. Front. Physiol. 8, 603. 10.3389/fphys.2017.00603 29075195 PMC5641583

[B25] Goswami N. Batzel J. J. Clément G. Stein T. P. Hargens A. R. Sharp M. K. (2013). Maximizing information from space data resources: A case for expanding integration across research disciplines. Eur. J. Appl. Physiol. 113 (7), 1645–1654. 10.1007/s00421-012-2507-5 23073848

[B26] Goswami N. Blaber A. P. Hinghofer-Szalkay H. Convertino V. A. (2019). Lower body negative pressure: Physiological effects, applications, and implementation. Physiol. Rev. 99 (1), 807–851. 10.1152/physrev.00006.2018 30540225

[B27] Goswami N. Blaber A. P. Hinghofer-Szalkay H. Montani J.-P. (2017). Orthostatic intolerance in older persons: Etiology and countermeasures. Front. Physiol. 8, 803. 10.3389/fphys.2017.00803 29163185 PMC5677785

[B28] Goswami N. Kavcic V. Marusic U. Simunic B. Rössler A. Hinghofer-Szalkay H. (2015). Effect of computerized cognitive training with virtual spatial navigation task during bed rest immobilization and recovery on vascular function: A pilot study. Clin. Interv. Aging 10, 453–459. 10.2147/CIA.S76028 25709419 PMC4330037

[B29] Goswami N. Roma P. G. De Boever P. Clément G. Hargens A. R. Loeppky J. A. (2012). Using the Moon as a high-fidelity analogue environment to study biological and behavioral effects of long-duration space exploration. Planet. Space Sci. 74 (1), 111–120. 10.1016/j.pss.2012.07.030

[B30] Grinsted A. Moore J. C. Jevrejeva S. (2004). Application of the cross wavelet transform and wavelet coherence to geophysical time series. Nonlinear process. geophys. 11, 561–566. 10.5194/npg-11-561-2004

[B31] Hargens A. R. Vico L. (2016). Long-duration bed rest as an analog to microgravity. J. Appl. Physiol. 120 (8), 891–903. 10.1152/japplphysiol.00935.2015 26893033

[B32] Hedge E. T. Patterson C. A. Mastrandrea C. J. Sonjak V. Hajj-Boutros G. Faust A. (2022). Implementation of exercise countermeasures during spaceflight and microgravity analogue studies: Developing countermeasure protocols for a bedrest in older adults (BROA). Front. Physiology 13, 1443. 10.3389/fphys.2022.928313 PMC9395735 36017336

[B33] Hermens H. J. Freriks B. Merletti R. Stegeman D. Blok J. Rau G. (1999). European recommendations for surface electromyography. Roessingh Res. Dev. 8 (2), 13–54.

[B34] Hirayanagi K. Iwase S. Kamiya A. Sasaki T. Mano T. Yajima K. (2004). Functional changes in autonomic nervous system and baroreceptor reflex induced by 14 days of 6 degrees head-down bed rest. Eur. J. Appl. Physiol. 92 (1), 160–167. 10.1007/s00421-004-1067-8 15042373

[B35] Hughson R. L. Shoemaker J. K. Blaber A. P. Arbeille P. Greaves D. K. Pereira-Junior P. P. (2012). Cardiovascular regulation during long-duration spaceflights to the international space station. J. Appl. Physiol. 112 (5), 719–727. 10.1152/japplphysiol.01196.2011 22134699

[B36] Iwasaki K.-I. Zhang R. Zuckerman J. H. Pawelczyk J. A. Levine B. D. (2000). Effect of head-down-tilt bed rest and hypovolemia on dynamic regulation of heart rate and blood pressure. Am. J. Physiol. Regul. Integr. Comp. Physiol. 279 (6), R2189–R2199. 10.1152/ajpregu.2000.279.6.R2189 11080085

[B37] Izumi Y. Matsumoto K. Ozawa Y. Kasamaki Y. Shinndo A. Ohta M. (2007). Effect of age at menopause on blood pressure in postmenopausal women. Am. J. Hypertens. 20 (10), 1045–1050. 10.1016/j.amjhyper.2007.04.019 17903686

[B38] Kortebein P. Ferrando A. Lombeida J. Wolfe R. Evans W. J. (2007). Effect of 10 days of bed rest on skeletal muscle in healthy older adults. Jama 297 (16), 1772–1774. 10.1001/jama.297.16.1772-b 17456818

[B39] Kwak S. K. Kim J. H. (2017). Statistical data preparation: Management of missing values and outliers. Korean J. Anesthesiol. 70 (4), 407–411. 10.4097/kjae.2017.70.4.407 28794835 PMC5548942

[B40] Lanier J. B. Mote M. B. Clay E. C. (2011). Evaluation and management of orthostatic hypotension. Am. Fam. Physician 84 (5), 527–536. 21888303

[B41] Laws J. Caplan N. Bruce C. McGrogan C. Lindsay K. Wild B. (2020). Systematic review of the technical and physiological constraints of the Orion Multi-Purpose Crew Vehicle that affect the capability of astronauts to exercise effectively during spaceflight. Acta Astronaut. 170, 665–677. 10.1016/j.actaastro.2020.02.038

[B42] LeBlanc A. D. Spector E. R. Evans H. J. Sibonga J. D. (2007). Skeletal responses to space flight and the bed rest analog: A review. J. Musculoskelet. Neuronal Interact. 7 (1), 33–47. 17396004

[B43] Lee S.-J. Lehar A. Meir J. U. Koch C. Morgan A. Warren L. E. (2020). Targeting myostatin/activin A protects against skeletal muscle and bone loss during spaceflight. Proc. Natl. Acad. Sci. U. S. A. 117 (38), 23942–23951. 10.1073/pnas.2014716117 32900939 PMC7519220

[B44] Mechling H. Netz Y. (2009). Aging and inactivity—Capitalizing on the protective effect of planned physical activity in old age. Eur. Rev. Aging Phys. Act. 6 (2), 89–97. 10.1007/s11556-009-0052-y

[B45] Moore A. D. Lee S. M. Stenger M. B. Platts S. H. (2010). Cardiovascular exercise in the US space program: Past, present and future. Acta astronaut. 66 (7-8), 974–988. 10.1016/j.actaastro.2009.10.009

[B46] Möstl S. Orter S. Hoffmann F. Bachler M. Hametner B. Wassertheurer S. (2021). Limited effect of 60-days strict head down tilt bed rest on vascular aging. Front. Physiol. 12, 685473. 10.3389/fphys.2021.685473 34122149 PMC8194311

[B47] Platts S. H. Martin D. S. Stenger M. B. Perez S. A. Ribeiro L. C. Summers R. (2009). Cardiovascular adaptations to long-duration head-down bed rest. Aviat. Space Environ. Med. 80 (5), A29–A36. 10.3357/asem.br03.2009 19476167

[B48] Ploutz-Snyder L. L. Downs M. Goetchius E. Crowell B. English K. L. Ploutz-Snyder R. (2018). Exercise training mitigates multisystem deconditioning during bed rest. Med. Sci. Sports Exerc. 50 (9), 1920–1928. 10.1249/MSS.0000000000001618 29924746 PMC6647016

[B49] Rapp K. Becker C. Cameron I. König H. Büchele G. (2012). Epidemiology of falls in residential aged care. J. Am. Med. Dir. Assoc. 13, 1. 21816682 10.1016/j.jamda.2011.06.011

[B50] Redfern M. S. Furman J. M. Jacob R. G. (2007). Visually induced postural sway in anxiety disorders. J. Anxiety Disord. 21 (5), 704–716. 10.1016/j.janxdis.2006.09.002 17045776 PMC1975822

[B51] Rice J. E. (2019). Orion spacecraft. Mitchell Lane.

[B52] Rodriguez J. Blaber A. P. Kneihsl M. Trozic I. Ruedl R. Green D. A. (2017). Poststroke alterations in heart rate variability during orthostatic challenge. Medicine 96, e5989. 10.1097/MD.0000000000005989 28383399 PMC5411183

[B53] Schneider S. M. Lee S. M. Feiveson A. H. Watenpaugh D. E. Macias B. R. Hargens A. R. (2016). Treadmill exercise within lower body negative pressure protects leg lean tissue mass and extensor strength and endurance during bed rest. Physiol. Rep. 4 (15), e12892. 10.14814/phy2.12892 27495299 PMC4985554

[B54] Scott J. P. Weber T. Green D. A. (2019). Introduction to the Frontiers research topic: Optimization of exercise countermeasures for human space flight–lessons from terrestrial physiology and operational considerations. Front. Physiol. 10, 173. 10.3389/fphys.2019.00173 30899226 PMC6416179

[B55] Sibonga J. D. Spector E. R. Johnston S. L. Tarver W. J. (2015). Evaluating bone loss in ISS astronauts. Aerosp. Med. Hum. Perform. 86 (12), A38–A44. 10.3357/AMHP.EC06.2015 26630194

[B56] Spector E. R. Smith S. M. Sibonga J. D. (2009). Skeletal effects of long-duration head-down bed rest. Aviat. Space Environ. Med. 80 (5), A23–A28. 10.3357/asem.br02.2009 19476166

[B57] Speers R. A. Paloski W. H. Kuo A. D. (1998). Multivariate changes in coordination of postural control following spaceflight. J. Biomech. 31 (10), 883–889. 10.1016/s0021-9290(98)00065-7 9840753

[B58] Sugihara G. May R. Ye H. Hsieh C.-h. Deyle E. Fogarty M. (2012). Detecting causality in complex ecosystems. science 338 (6106), 496–500. 10.1126/science.1227079 22997134

[B59] Swain P. Laws J. De Martino E. Wotring V. Caplan N. Winnard A. (2021). Effectiveness of exercise countermeasures for the prevention of musculoskeletal deconditioning in simulated hypogravity: A systematic review. Acta Astronaut. 185, 236–243. 10.1016/j.actaastro.2021.05.005

[B60] Taddei S. (2009). Blood pressure through aging and menopause. Climacteric 12 (1), 36–40. 10.1080/13697130903004758 19811239

[B61] Team R. C. (2011). R development core team. R: A language and environment for statistical computing. Vienna: R Foundation for Statistical Computing.

[B62] Tikhonoff V. Casiglia E. Gasparotti F. Spinella P. (2019). The uncertain effect of menopause on blood pressure. J. Hum. Hypertens. 33 (6), 421–428. 10.1038/s41371-019-0194-y 30899074

[B63] Traon P.-L. Heer M. Narici M. V. Rittweger J. Vernikos J. PAvy-Le TrAon A. (2007). From space to Earth: Advances in human physiology from 20 years of bed rest studies (1986–2006). Eur. J. Appl. Physiol. 101 (2), 143–194. 10.1007/s00421-007-0474-z 17661073

[B64] Traon P.-L. Vasseur P. Sigaudo D. Maillet A. Fortrat J. Hughson R. (1997). Cardiovascular responses to orthostatic tests after a 42-day head-down bed-rest. Eur. J. Appl. Physiol. Occup. Physiol. 77 (1), 50–59. 10.1007/s004210050299 9459521

[B65] Trozic I. Platzer D. Fazekas F. Bondarenko A. I. Brix B. Rössler A. (2020). Postural hemodynamic parameters in older persons have a seasonal dependency : A pilot study. Z. Gerontol. Geriatr. 53 (2), 145–155. 10.1007/s00391-019-01525-3 30868225 PMC7066096

[B66] Vandewoude M. F. Alish C. J. Sauer A. C. Hegazi R. A. (2012). Malnutrition-sarcopenia syndrome: Is this the future of nutrition screening and assessment for older adults? J. aging Res. 2012, 651570. 10.1155/2012/651570 23024863 PMC3449123

[B67] Verheyden B. Beckers F. Couckuyt K. Liu J. Aubert A. (2007). Respiratory modulation of cardiovascular rhythms before and after short-duration human spaceflight. Acta Physiol. 191 (4), 297–308. 10.1111/j.1748-1716.2007.01744.x 17784903

[B68] Verma A. K. Garg A. Xu D. Bruner M. Fazel-Rezai R. Blaber A. P. (2017a). Skeletal muscle pump drives control of cardiovascular and postural systems. Sci. Rep. 7 (1), 45301–45308. 10.1038/srep45301 28345674 PMC5366896

[B69] Verma A. K. Xu D. Bruner M. Garg A. Goswami N. Blaber A. P. (2018). Comparison of autonomic control of blood pressure during standing and artificial gravity induced via short-arm human centrifuge. Front. Physiol. 9, 712. 10.3389/fphys.2018.00712 29988521 PMC6026653

[B70] Verma A. K. Xu D. Garg A. Blaber A. P. Tavakolian K. (2019). Effect of aging on muscle-pump baroreflex of individual leg muscles during standing. Front. Physiol. 10, 845. 10.3389/fphys.2019.00845 31379591 PMC6646886

[B71] Verma A. K. Xu D. Garg A. Cote A. T. Goswami N. Blaber A. P. (2017b). Non-linear heart rate and blood pressure interaction in response to lower-body negative pressure. Front. Physiol. 8, 767. 10.3389/fphys.2017.00767 29114227 PMC5660688

[B72] Veronese N. De Rui M. Bolzetta F. Zambon S. Corti M. C. Baggio G. (2015). Orthostatic changes in blood pressure and mortality in the elderly: The pro. VA study. Am. J. Hypertens. 28 (10), 1248–1256. 10.1093/ajh/hpv022 25767137

[B73] Wang J. Song Y. Gao M. Bai X. Chen Z. (2016). Neuroprotective effect of several phytochemicals and its potential application in the prevention of neurodegenerative diseases. Geriatrics 1 (4), 29. 10.3390/geriatrics1040029 PMC6371135 31022822

[B74] Xu D. Tremblay M. F. Verma A. K. Tavakolian K. Goswami N. Blaber A. P. (2020). Cardio-postural interactions and muscle-pump baroreflex are severely impacted by 60-day bedrest immobilization. Sci. Rep. 10 (1), 1–13. 10.1038/s41598-020-68962-8 32694819 PMC7374578

[B75] Xu D. Verma A. K. Garg A. Bruner M. Fazel-Rezai R. Blaber A. P. (2017). Significant role of the cardiopostural interaction in blood pressure regulation during standing. Am. J. Physiol. Heart Circ. Physiol. 313 (3), H568–H577. 10.1152/ajpheart.00836.2016 28626082 PMC5625169

