# Supplementary material for: Canadian aging and inactivity study: Spaceflight-inspired exercises during head-down tilt bedrest blunted reductions in muscle-pump but not cardiac baroreflex in older persons
Source: Front Physiol. 2022 Sep 21;13:943630. doi: 10.3389/fphys.2022.943630 (PMC9532525; doi:10.3389/fphys.2022.943630)
Supplement: Supplementary file 1 [file DataSheet1.docx]

**Canadian aging and inactivity study: Spaceflight-inspired exercises during head-down tilt bedrest blunted reductions in muscle-pump but not cardiac baroreflex in older persons**

SUPPLEMENTAL MATERIAL

**Supplemental Methods**

Selection Criteria

**Inclusion criteria for participating:**

- A minimum of 20 and a maximum of 24 non-smoking participants in the age group of 55 to 65 years old, half male and half female.
- Female participants must be menopausal (no menses for at least 1 year (or documented ovariectomy) and a serum FSH above 30 IU/L). Most women aged 55-65 are menopausal. In case any of them are not, they will need to be excluded. This is because having menopausal and non-menopausal women, would lead to a greater variability and we would not be able to accurately compare women between the intervention and control groups.
- Height between 158 to 190 cm with a body mass index between 20 to 30 kg/m^2^.
- Physically and mentally healthy subjects that will have successfully passed the psychological and medical screening appropriate for the age group.
- Sedentary people and people that are addicted to exercise are excluded. Participants will include those that participate in at least 2.5 hours of exercise at a moderate to vigorous-intensity aerobic activity per week.
- Willing to be assigned randomly either to the exercise or the control group.

**Exclusion criteria for participating:**

- Participants must be dementia-free, drug- or alcohol-addiction free, with no history of heart attacks, no thrombosis risk, no severe allergies, no hypocalcemia, no uric acidemia, no orthostatic intolerance, no vestibular disorders, no considerable musculoskeletal issues, no chronic back pain, no head trauma, no seizures, no ulcers, no renal stones, no gastro-esophageal reflux disease or renal function disorder, no hiatus hernia, no migraines, and no mental illness. These conditions can occur during prolonged bedrest and participants already presenting these conditions may not be able to safely complete the study. Their participation could even worsen their condition.
- Applicants who cannot conform to the prescribed diet, or who object to frequent blood collection will be excluded.
- Participants must not have:
  - Electrocardiogram abnormalities
  - HIV – This is standard practice as this study involves blood and tissue handling
  - Anemia - Ferritin range outside 10 to 154 ng/ml (Females); 20 to 245 ng/ml (Males). This is because participants will undergo blood collection at various timepoints throughout the study
  - A family history of thrombosis
  - Bone mineral density (measured by DEXA) greater than 2.0 standard deviation ≤ t-score
  - Medication requirements that may interfere with the interpretation of the results. No blood pressure medication allowed
  - Recent substandard nutritional status
  - Claustrophobia
  - Special dietary requests (e.g., vegetarian, vegan, or some other diet)
  - Metallic implants (pacemakers, ICDs, CRT devices, infusion pumps, cerebral artery aneurysm clips, dental implants, tissue expander etc.), osteosynthesis material
  - Given blood in the past 3 months before the onset of the experiment
  - Smoked within 6 months prior to the start of the study
  - Abused drugs, medicine, or alcohol within up to 30 days prior to the start of the study
  - Participated in another study within 2 months before study onset
  - A criminal record or have been incarcerated. This is because of safety issues as these individuals may have personality disorders. Additionally, this is a government funded study who requires a high level of security and data protection
  - A positive COVID-19 test taken 1 week to 24h before study start date

**Rules for behavior during bedrest**

- All bed rest participants were given the following instructions as standard procedures and requirements, which were also mentioned in the ethical protocols. The European Space Agency has used similar approaches in prior bed rest research [1].
- Participants were required to follow a day-night cycle that began at 7 a.m. and ended at 11 p.m. Participants were instructed that they could change positions in bed during bed rest, but that physical activity should be kept to a minimal and that all hygiene should be done in the head-down tilt position. Showering was done in a specially designed head-down tilt shower bed. Participants were allowed to lie on their stomach, back, or side, but they were informed that when resting on their side, their trunk and head had to be in a head-down posture (i.e., the head couldn't be raised up with their hand/arm as one might do when reading). Alarm monitoring was carried out 24 hours a day to ensure that participants followed the research procedure.
- Throughout the baseline, individuals were not permitted to nap or lie on their beds during the day (BDC).
- It was supposed that the participants had to finish their tray meals.
- They are not permitted to leave the institution unless for specific measures (MRI, for example), and even then, they must be accompanied by a member of the staff.
- They were expected to adhere to the study's and scientific testing' schedules. During the tests, they had to follow the directions of the personnel and the scientists.
- They were permitted to make phone calls, but they couldn't interfere with the scientific studies or other operations.

**Study diet and dietary supplementation**

Participants will receive a weight-maintaining diet throughout the study. As their resting metabolic rate (RMR) will be measured by indirect calorimetry during the baseline period, their energy intake will match RMR x 1.1 to which a surplus of 10% is added to compensate for the thermic effect of food as proposed by the ISA which should be close to measured RMR x1.2. During the baseline and recovery phases their energy intake will be set at RMR x 1.5, including the thermic effect of food. Energy intake of participants in the exercise group will be corrected by the energy expenditure of exercise that will be measured by an indirect portable calorimeter during their preparation session of the baseline phase, meaning that those participants will consume more calories per day on exercise day. The daily menu will be composed of high-quality protein value and intake appropriate for this age-group of 1.2 g /kg/day with at least 0.4 g/kg/meal with a balanced macronutrient content with vitamins and minerals. They will receive a multivitamin and mineral preparation to meet pre-established calcium intake of 1200 mg and vitamin D of 1000 IU per day. Since energy expenditure will decrease due to bedrest, some may end up with fairly low energy intakes (≤1500, women especially); they may need some protein-enriched foods and/or supplements to reach the protein intake goal. All food ingested during the 26-day inpatient stay will be monitored using the software Keenoa (a phone application that recognizes food items from a picture), to control for caloric and macronutrient intake. During this period participants will be asked to fill out a food intake and satiety questionnaire. Additionally, at the beginning of the study and follow-up visits participants will also be asked to fill out food frequency questionnaire and to track their nutrient intake for 3 days with the Keenoa application. In case any of the participants do not have a phone with a camera, they will be provided with a hard copy of the 3-day food diary. On one day in baseline and one day in HDBR, blood samples will be obtained 1 h prior to and 1 h after completing the meal to measure appetite related hormones.

**Data Analysis**

The electrocardiogram (ECG) was used to create a time series of heartbeat period (i.e., RR-interval) and heart rate (HR). The continuous BP during each RR-interval was used to calculate the systolic blood pressure (SBP). To reflect overall muscle activity, aggregate electromyography (EMG) was created by adding rectified EMG signals from all individual leg muscles. A moving average filter was used to capture the EMG envelope. Finally, the EMG impulse (EMGimp) was determined as the area under the aggregate EMG envelope during each heartbeat to indicate the muscular contraction strength on a beat-by-beat basis, analogous to the impulse of force. The idea of impulse was used since the strength of muscle contraction over a heartbeat would be linked to the length of that beat on a beat-by-beat basis. That is, a powerful contraction for a short time can be deemed comparable to lesser contractions over a longer time, and the same contraction intensity would result in greater total strength over a longer heartbeat. Prior to the wavelet transform and causality analysis, the beat-by-beat time series were resampled to 10 Hz using spline interpolation. Data analysis was described in detail by Xu et al. (Xu et al., 2017).

*Wavelet transform coherence*: For the signal pair SBP→EMGimp (muscle-pump baroreflex), the Morlet wavelet was used to create time-frequency distributions of wavelet transform coherence (WTC) (Garg et al., 2013; Garg et al., 2014). The Monte Carlo method was used to determine the substantial coherence threshold (Xu et al., 2017). This research looked at three frequency bands: very low frequency (VLF, 0.03–0.07 Hz), low frequency (LF, 0.07–0.15 Hz), and high frequency (HF, 0.15–0.5 Hz). There were 49 sub-band segments created from the three frequency bands (15 in VLF, 13 in LF, and 21 in HF) (Xu et al., 2017). The area over the substantial coherence threshold in each frequency band was divided by the entire area of that frequency band to calculate the fraction time active (FTA). The response gain was measured by averaging the cross wavelet transforms of the two signals (Grinsted et al., 2004) over significant WTC areas within each frequency band.

*Cause-and-effect relationship*: The convergent cross mapping (CCM) approach (Sugihara et al., 2012) was used to calculate the statistical correlation between signal pairs of interest (EMGimp↔SBP). By analyzing the correlation coefficient between the original signal and its estimate, the intensity of causal direction in the forward and backward directions was assessed (Verma et al., 2017). The time delay and embedding dimension were used to create the manifold of each signal. Based on the minimization of false nearest neighbor at a delay of 10 samples, the ideal dimension of reconstruction for cardio-postural signals to capture physiological variations within a heartbeat range was determined to be 4. Verma et al. (Verma et al., 2017) and Sugihara et al. supplemental's material (Sugihara et al., 2012) have more information on the technique.

*Gain, FTA and Causality Relationship*: To build a reflex efficiency indicator, we choose to investigate the interplay between gain, FTA, and causality. Given the observed changes in both gain and FTA, it's reasonable to assume that a genuine evaluation of the system's efficacy is a function of both. The interaction can only be used while the two systems are interacting, which is indicated in the fraction time active determination. The operating gain, in other words, is only beneficial during the time of active interaction. As a result, we invented the phrase "Active" Gain, which is the product of the two values (Gain×FTA). Causality (between 0 and 1) is a measure of the intensity of the signal directionality, with a lower value suggesting a weaker causal association. To visualize this interaction in connection to the muscle-pump baroreflex and HDBR, a two-dimensional plot (Active Gain versus Causality) was created.

**References**

Garg, A., Xu, D., and Blaber, A.P. (2013). Statistical validation of wavelet transform coherence method to assess the transfer of calf muscle activation to blood pressure during quiet standing. *Biomedical engineering online* 12(1)**,** 1-14.

Garg, A., Xu, D., Laurin, A., and Blaber, A.P. (2014). Physiological interdependence of the cardiovascular and postural control systems under orthostatic stress. *American journal of physiology-Heart and Circulatory Physiology* 307(2)**,** H259-H264.

Grinsted, A., Moore, J.C., and Jevrejeva, S. (2004). Application of the cross wavelet transform and wavelet coherence to geophysical time series. *Nonlinear processes in geophysics* 11(5/6)**,** 561-566.

Sugihara, G., May, R., Ye, H., Hsieh, C.-h., Deyle, E., Fogarty, M., et al. (2012). Detecting causality in complex ecosystems. *science* 338(6106)**,** 496-500.

Verma, A.K., Garg, A., Xu, D., Bruner, M., Fazel-Rezai, R., Blaber, A.P., et al. (2017). Skeletal muscle pump drives control of cardiovascular and postural systems. *Scientific Reports* 7(1)**,** 1-8.

Xu, D., Verma, A.K., Garg, A., Bruner, M., Fazel-Rezai, R., Blaber, A.P., et al. (2017). Significant role of the cardiopostural interaction in blood pressure regulation during standing. *American Journal of Physiology-Heart and Circulatory Physiology* 313(3)**,** H568-H577.
